# 16S-ARDRA and MALDI-TOF mass spectrometry as tools for identification of *Lactobacillus* bacteria isolated from poultry

**DOI:** 10.1186/s12866-016-0732-5

**Published:** 2016-06-13

**Authors:** Marta Dec, Andrzej Puchalski, Renata Urban-Chmiel, Andrzej Wernicki

**Affiliations:** Sub-Department of Veterinary Prevention and Avian Diseases, Institute of Biological Bases of Animal Diseases, Faculty of Veterinary Medicine, University of Life Sciences in Lublin, Akademicka 12, 20-033 Lublin, Poland

**Keywords:** *Lactobacillus*, Lactic acid bacteria, Identification, Poultry, MALDI-TOF MS, ARDRA, 16S rDNA

## Abstract

**Background:**

The objective of our study is to evaluate the potential use of Amplified 16S Ribosomal DNA Restriction Analysis (16S-ARDRA) and MALDI-TOF mass spectrometry (MS) as methods for species identification of *Lactobacillus* strains in poultry.

**Results:**

A total of 80 *Lactobacillus* strains isolated from the cloaca of chicken, geese and turkeys were identified to the species level by MALDI-TOF MS (on-plate extraction method) and 16S-ARDRA. The two techniques produced comparable classification results, some of which were additionally confirmed by sequencing of 16S rDNA. MALDI-TOF MS enabled rapid species identification but produced more than one reliable identification result for 16.25 % of examined strains (mainly of the species *L. johnsonii*). For 30 % of isolates intermediate log(scores) of 1.70–1.99 were obtained, indicating correct genus identification but only presumptive species identification. The 16S-ARDRA protocol was based on digestion of 16S rDNA with the restriction enzymes *Mse*I, *Hinf*I, *Mbo*I and *Alu*I. This technique was able to distinguish 17 of the 19 *Lactobacillus* reference species tested and enabled identification of all 80 wild isolates. *L. salivarius* dominated among the 15 recognized species, followed by *L. johnsonii* and *L. ingluviei*.

**Conclusions:**

The MALDI-TOF MS and 16S-ARDRA assays are valuable tools for the identification of avian lactobacilli to the species level. MALDI-TOF MS is a fast, simple and cost-effective technique, and despite generating a high percentage of results with a log(score) <2.00, the on-plate extraction method is characterized by high-performance. For samples for which Biotyper produces more than one reliable result, MALDI-TOF MS must be used in combination with genotypic techniques to achieve unambiguous results. 16S-ARDRA is simple, repetitive method with high power of discrimination, whose sole limitation is its inability to discriminate between species with very high 16S rDNA sequence homology, such as *L. casei* and *L. zeae*. The assays can be used for discrimination of *Lactobacillus* bacteria from different habitats.

## Background

Lactobacilli are Gram-positive, non-sporing, aerotolerant or anaerobic catalase-negative rods or coccobacilli. The genus *Lactobacillus* currently (December 2015) comproses 224 species [1] and is thus the most numerous group of lactic acid bacteria (LAB). The natural habitats of these bacteria are dairy products, healthy and rotting plants, and the mucous membranes of humans and animals, including birds. They have been isolated from the GIT (gastrointestinal tract) of chickens [2], geese [3], ducks [4] and pigeons [5]. The most commonly identified species in these birds are *L. salivarius*, *L. johnsonii*, *L. crispatus*, *L. reuteri* and *L. agilis* [2–5].

Lactobacilli, as beneficial components of the gut microbiome, have a great impact on the health status of farm animals, including poultry. While maintaining the microbial balance of the mucous membranes, they provide protection against enteropathogenic infection [6, 7]. In addition, they improve digestion and nutrient assimilation, remove toxic substances, and enhance immunity [8, 9]. Owing to their health-promoting properties *Lactobacillus* bacteria are used to produce probiotic preparations for humans and animals. Probiotics, through multi-pronged action, improve the health of animals and increase the efficiency of livestock production. Interest in the application of probiotics in poultry has grown since the introduction in the EU of a ban on antibiotic growth promoters in animals and the associated increase in the frequency of intestinal infections in birds, mainly induced by *C. perfringens*. The use of selected *Lactobacillus* strains as feed additives for poultry can produce similar effects to those of antibiotic growth promoters, manifested by increases in weight and better feed efficiency [10, 11], as well as resistance to pathogenic bacteria such as *Salmonella* sp. [12], *C. perfringens* [13, 14], *E. coli* [10] or *Campylobacter* sp. [14]. Moreover, supplementing the diet of broilers with *Lactobacillus* strains reduces fat deposition in the coelom [15] and increases the size, quality and production of eggs [16, 17].

Accurate taxonomic classification of lactobacilli to the species level is not an easy task. It is made difficult by the large and continually growing number of species belonging to this genus and their biochemical and genetic diversity. Identification by phenotypic methods is time-consuming and has a low discriminatory level [18]. The commercial kit API CHL50 (Biomerieux) for lactic acid bacilli yields ambiguous results and even misidentifications [19]. Molecular methods have proven to be more reliable. The target most commonly used for bacterial identification is 16S rDNA. This ~1500 base-pair gene is characterized by slow rates of evolution and encodes 16S rRNA, a component of the 30S small subunit of prokaryotic ribosomes. In addition to highly conserved sites (used for binding of universal primers in PCR), 16S rRNA gene sequences contain hypervariable regions that can provide species-specific signature sequences useful in identifying bacteria and determining their phylogenetic position [20]. Despite its accuracy, the use of 16S rRNA gene sequence analysis is not widespread outside of reference laboratories because of technical and cost considerations. Sequencer purchase prices exceed the financial capacity of ordinary laboratories, and the costs of sequencing performed by outside labs offering this service is not cost-effective for identification of multiple strains. The high price (about €30 per sample) is dictated by the substantial length of 16S rDNA, which requires two sequencing reactions (automated Sanger dideoxy method) and assembly of the two fragments using appropriate software.

Another method for identifying bacteria, based on analysis of the gene encoding 16S rRNA, is Amplified Ribosomal DNA Restriction Analysis of 16S rDNA (16S-ARDRA). It is a simple method that can be routinely used in laboratories because it does not require specialized equipment. It is also less expensive than 16S rDNA sequencing (costs of identification depend primarily on the price of reference strains and restriction enzymes). The power of discrimination of ARDRA depends on the restriction enzymes used, which can be selected on the basis of *in silico* analysis using 16S rDNA sequences accumulated in public databases. Strains are identified by comparing the electrophoretic profiles of restriction fragments of wild-type strains with profiles of reference strains [21].

Matrix-assisted laser desorption/ionization time-of-flight mass spectrometry (MALDI-TOF MS) is an increasingly used technique enabling quick identification of isolates to the species or even sub-species level. It is a valuable alternative to more time-consuming and more expensive methods, including 16S rRNA gene sequencing and 16S-ARDRA, which require DNA extraction, amplification and electrophoretic separation [22, 23]. During MALDI-TOF MS, microbes are identified using either intact cells or cell extracts, chemical compounds are ionized into charged molecules, and their mass-to-charge ratio (m/z) is measured. This technology generates mass spectra mostly composed of highly abundant proteins, including many ribosomal proteins assumed to be characteristic for each bacterial species. Mass spectra based on detected proteins are unique signatures which are treated as a fingerprint of the sample. The identification relies on comparison of the mass spectrum of the tested isolate with those of strains in reference databases [23]. The reliability of the identification results obtained by MALDI-TOF MS is comparable to that of genetic typing methods, including 16S rDNA sequencing [3, 22, 24, 25]. The main limitation of the technology is that identification of new isolates is possible only if the spectral database contains peptide mass fingerprints of the type strains of specific genera, species or subspecies.

In the present study, MALDI-TOF MS and 16S-ARDRA were evaluated as methods for identification of *Lactobacillus* bacteria isolated from poultry, including chicken, geese and turkeys.

## Methods

### Bacteria and growth conditions

*Lactobacillus* isolates were collected from the fresh faeces or cloacae of 12 White Koluda geese, 10 broilers and 3 turkeys from large-scale poultry farms in Poland.

A total of 89 bacterial strains, including 46 strains from geese, 35 from broilers and 9 from turkeys, were isolated on MRS (Man, Rogosa and Sharp) medium (BTL, Poland) supplemented with 0.05 % (w/v) cysteine hydrochloride (Sigma-Aldrich, Poland) (MRS-cys) at 37 °C for 48 h in 5 % CO_2_. All isolates were Gram-positive and catalase-negative. There were 10 strains (8 strains of goose origin and 2 of chicken origin) with coccus morphology that were excluded from further analysis. A total of 80 isolates (38 from geese, 33 from chickens and 9 from turkeys) with rod-shaped morphology were considered to be lactobacilli and were stored at −80 °C until further analysis. A total of 23 reference *Lactobacillus* strains, listed in Table 1, were obtained from the BCCM™/LMG bacteria collection (Ghent, Belgium) or from Argenta (Poland).

**Table 1:** Reference strains tested by MALDI-TOF MS analysis. The two best matches obtained in the Biotyper were taken into account. For strains for which the first and second best match indicated the same species, only one result (with the highest log(score)) is shown in the table. A-U – symbols for reference strains used in Table 3 and in Figs. 1, 2, 3 and 4

No.	Reference strains		MALDI-TOF MS	Biotyper log(score)
	Species	Strain number		
1	*L. acidophilus* (F)	ATCC 4356	*L. acidophilus*	2.296
2	*L. agilis* (C)	LMG 9186	*L. agilis*	2.321
3	*L. amylovorus* (J)	LMG 9496	*L. amylovorus*	2.351
4	*L. casei* (M)	ATCC 393	*L. zeae*	1.954
			*L. casei*	1.84
5	*L. crispatus* (I)	LMG 9479	*L. crispatus*	2.117
6	*L. farciminis* (E)	LMG 9189	*L. farciminis*	1.982
7	*L. gallinarum* (L)	LMG 9435	*L. gallinarum*	2.179
			*L. acidophilus*	1.965
8	*L. gasseri* (G)	ATCC 19992	*L. gasseri*	2.316
9	*L. ingluviei* (R1)	LMG 20380	*L. ingluviei*	1.957
10	*L. ingluviei* (R2)	LMG 22056	*L. ingluviei*	1.881
11	*L. johnsonii* (H)	LMG 9436	*L. johnsonii*	1.994
			*L. gasseri*	1.897
12	*L. kitasatonis* (K)	LMG 23133	*L. kitasatonis*	2.078
			*L. amylovorus*	1.953
13	*L. mucosae* (S)	*LMG* 19534	*L. mucosae*	2.023
14	*L. oris* (U)	LMG 9848	*L. oris*	1.949
			*L. antri*	1.79
15	*L. plantarum* (D)	ATCC 8014	*L. plantarum*	2.002
16	*L. paracasei* (P)	ATCC BAA-52	*L. paracasei*	2.133
17	*L. reuteri* (T1)	LMG 9213	*L. reuteri*	2.192
18	*L. reuteri* (T2)	LMG 18238	*L. reuteri*	1.971
19	*L. rhamnosus* (N)	ATCC 7469	*L. rhamnosus*	2.324
20	*L. saerimneri* (B)	LMG 22875	*L. saerimneri*	2.249
21	*L. salivarius* (A1)	LMG 9476	*L. salivarius*	2.224
22	*L. salivarius* (A2)	LMG 9477	*L. salivarius*	2.342
23	*L. zeae* (O)	LMG 17315	*L. casei*	2.021
			*L. zeae*	2.007

### Species identification using MALDI-TOF MS

Measurements were performed with a UltrafleXtreme MALDI TOF mass spectrometer (Bruker, Germany) equipped with a 1000 Hz Nd-YAG laser (neodymium-doped yttrium aluminium garnet) as it was descriped in our previous work. In a simple direct method, a single bacterial colony grown on MRS agar was transferred onto a spot of the 384 MTP AnchorChip™ T F stainless steel MALDI target plate (Bruker, Germany). Subsequently, the bacterial sample was overlaid with 1 μL 70 % formic acid and then with 1 μL matrix solution containing 10 mg/mL HCCA (a-cyano-4-hydroxycinnamic acid, Sigma-Aldrich, Poland) resolved in 50 % acetonitrile (Sigma-Aldrich, Poland) and 2.5 % TFA (trifluoro-acetic acid, Sigma-Aldrich, Poland) and air-dried [26, 27].

The MALDI target plate was then introduced into the spectrometer for automated measurement and data interpretation. Prior to the analyses, calibration was performed with a bacterial test standard (Bruker, Germany) containing extract of *E. coli* DH5 alpha.

The mass spectra were processed with the MALDI Biotyper 3.0 software package (Bruker, Germany) containing 3995 reference spectra, including 218 for lactobacilli. The results were shown as the top 10 identification matches along with confidence scores ranging from 0.00 to 3.00.

According to the criteria recommended by the manufacturer, a log(score) below 1.70 does not allow for reliable identification; a log(score) between 1.70 and 1.99 allows identification to the genus level; a log(score) between 2.00 and 2.29 means highly probable identification at the genus level and probable identification at the species level; and a log(score) higher than 2.30 (2.30 – 3.00) indicates highly probable identification at the species level.

Analysis of each sample was performed in triplicate (3 spots for each sample). If the log scores from the first run were <2.00 or a sample yielded a MALDI mass spectrum with no peaks a second run was performed.

The result of identification was considered reliable when at least the two best matches (log(score) 1.70-3.00) with the MALDI Biotyper database indicated the same species. For samples for which the top two matches indicated different species, we took into account the first match, provided that the log(score) was greater than the value for the second match of ≥0.30.

### Isolation of bacterial DNA

For DNA analysis the wild and reference *Lactobacillus* strains were grown in MRS-cys broth for 18 h and genomic DNA was isolated using a GeneMATRIX Bacterial & Yeast Genomic DNA Purification Kit (Eurx, Poland) following the manufacturer’s instructions with some modifications. Lysozyme (30 mg/mL, Sigma-Aldrich, Poland) and mutanolysin (30 U/mL, Sigma-Aldrich, Poland) were added to lysis buffer. Bacteria suspended in lysis buffer were incubated for 1.5 h at 37 °C. Further DNA isolation steps were performed according to the manufacturer’s protocol.

### Amplification of the 16S rRNA gene

The 16S rDNA gene was amplified by PCR using primers fD1 5′-AGA GTT TGA TCC TGG CTC AG-3′ and R1530 5′-AAG GAG GTG ATC CAG CCG CA-3′ [24] obtained from Blirt (Poland). The PCR reactions were performed in an Eppendorf Mastercycler in a 50 μL reaction mixture consisting of 25 μL DreamTaq PCR Master Mix (Thermo Scientific, USA), 3 μL of each primer (100 pmol/μL, Blirt, Poland), 3 μL of template DNA (~20 ng/μL) and 16 μL deionized water. The thermocycle programme was as follows: initial denaturation at 94 °C for 5 min; 30 cycles of 94 °C for 45 s, 56 °C for 45 s and 72 °C for 1.5 min; and a final extension step at 72 °C for 8 min. For all *Lactobacillus* strains tested, an ~1500-bp PCR single product was obtained.

### Digestion of 16S rDNA amplicons

In the preliminary tests using only *Lactobacillus* reference strains, the PCR products were digested with the following restriction enzymes: *Alu*I, *Mse*I, *Hae*III, *Mps*I, *Tag*I, *Hinf*I and *Mbo*I. These restriction enzymes were selected on the basis of *in silico* analysis using the nucleotide sequence of the whole 16S rDNA gene of different *Lactobacillus* strains, deposited in GenBank. Six μl of PCR product was digested in 12 μL of restriction enzyme buffer with 0.6 μL of restriction enzyme (initial concentration of each enzyme 10 U/μL) and left to react at 65 °C (for *Taq*I and *Mse*I) or at 37 °C (for *Alu*I, *Hae*III, *Mps*I, *Hinf*I and *Mbo*I) for 4 h. All restriction enzymes were purchased from Thermo Scientific (USA).

The restriction enzymes with the greatest discriminatory power for the reference lactobacilli, i.e., *Mse*I*, Hind*I*, Mbo*I and *Alu*I*,* were used to digest the PCR products obtained on the DNA matrix of the wild-type strains.

### DNA electrophoresis and analysis of restriction profiles

The DNA restriction fragments were separated by electrophoresis in a 3 % (wt/vol) high-resolution agarose (Prona) gel with ethidium bromide (0.5 mg/mL) in 0.5x Tris-borate-EDTA (pH 8.0) buffer at 90 V for 60 min and visualized under a UV source. Each gel was documented with a GelDoc apparatus (BioRad, USA). For all investigated bacterial strains (reference and wild), restriction fragment sizes were measured (in bp) by comparison with the M100-1000 bp DNA Ladder (Blirt, Poland) using Quantity One software (BioRad).

### Cluster analysis

To evaluate genetic diversity among the strains, the ARDRA profiles were analysed and used to construct a dendrogram. Each restriction fragment was treated as an individual character and scored as 1 (presence) or 0 (absence). The percent disagreement was used to cluster the isolates by the unweighted pair group mean arithmetic method (UPGMA) in Statistica 9.0 (StatSoft, Inc., Tulsa, USA). The results have been expressed for convenience as a percentage of similarity between the restriction profiles of the strains tested.

### 16S rDNA sequencing

PCR products from the 16S rRNA gene (~1500-bp) were purified with an ExoSap-IT kit (Affymetrix, USA) according to the supplier’s instructions. The DNA sequence was determined by a commercial DNA sequencing service provider (Genomed, Warsaw, Poland) using the same primers as those for PCR and Sanger method. Sequences were assembled with CLC Genomics Workbench 7.0 (CLC bio, a Qiagen Company) and compared to reference sequences available in the GenBank database using the NCBI BLAST algorithm (http://www.ncbi.nlm.nih.gov/BLAST).

## Results

### Identification of *Lactobacillus* strains using MALDI-TOF MS

#### Reference strains

As a preliminary test of the applicability of MALDI-TOF MS for the identification of *Lactobacillus* species, a set of 23 reference strains were analysed. The log(score) for 7 strains were between 1.70 and 1.99 (first best match), for 13 strains ranged from 2.00 to 2.29, and for 3 strains it was >2.3 (Table 1). Despite the many low values of log(score), that, according to Brucker’s criteria, allows only for identification to the genus level, the analysis yielded the correct results for all *Lactobacillus* strains. However, the identification of the 4 strains was considered as unreliable as the first best match was correct (except *L. casei*), and the second match of the similar values of log (score), pointed to another closely related species. Such equivocal results has been obtained in the case *L. casei* ATCC 393, *L. johnsonii* LMG 9436, *L. kitasatonis* LMG 23133 and *L. zeae* LMG 17315. Differences between log values (scores) of the first and second matching with those strains were ≤0.203.

### Wild isolates

A total of 80 isolates od rod-shaped morfology were classified as bacteria of the genus *Lactobacillus* with a Biotyper log (score) equal or greater than 1.70. For 7 (8.75 %) of the strains the log(score) was 2.3–3.0, for 49 (61.25 %) strains it was 2.00–2.29, and for 24 (30 %) it was 1.70–1.99.

For 67 (83.75 %) strains either at least the two best matches in Biotyper indicated the same species or the difference between the first and second best matches indicating different species was greater than 0.30. Identification of these isolates was considered to be reliable. For 13 samples the first and second best matches indicated different species, and the differences between their log(score) values were less than 0.22. Among these samples, for 12 isolates the best match indicated *L. johnsonii* and the second best match *L. gasseri*, and for one strain the best match indicated *L. kitasatonis* and second best match *L. amylovorus* (Table 2).

**Table 2:** Identification of wild *Lactobacillus* isolates by MALDI-TOF MS compared to the results obtained using 16S-ARDRA analysis

No. of strains	MALDI-TOF MS		16S-ARDRA (% similarity between wild and reference strains, based on Fig. 5)
	1st best match (1.70–3.00)	2nd best match (≤2.29)	
17	*L. salivarius*	*L. salivarius*	*L. salivarius* (100 %)
12	*L. johnsonii*	*L. gasseri*	*L. johnsonii* (100 %)
11	*L. ingluviei*	*L. ingluviei*	*L. ingluviei* (98.5 %)
8	*L. crispatus*	*L. crispatus*	*L. crispatus* (100 %)
8	*L. reuteri*	*L. reuteri*	*L. reuteri* (93.5 %)
4	*L. agilis*	*L. agilis*	*L. agilis* (98.5 %)
4	*L. oris*	*L. oris*	*L. oris* (97 %)
3	*L. plantarum*	*L. plantarum*	*L. plantarum* (98.5 %)
3	*L. paracasei*	*L. paracasei*	*L. paracasei* (100 %)
1	*L. rhamnosus*	*L. rhamnosus*	*L. rhamnosus* (100 %)
2	*L. amylovorus*	*L. amylovorus*	*L. amylovorus* (100 %)
1	*L. kitasatonis*	*L. amylovorus*	*L. kitasatonis* (100 %)
2	*L. farciminis*	*L. farciminis*	*L. farciminis* (91.5 %)
2	*L. saerimneri*	*L. saerimneri*	*L. saerimneri* (100 %)
Total 80			

Among the 80 strains identified to the species level (log(score) 1.7–3.0) were: *L. salivarius* – 17 strains, *L. johnsonii/L.gasseri* – 12, *L. ingluviei* – 11, *L. crispatus* – 8, *L. reuteri* – 8, *L. agilis* – 4, *L. oris* – 4,, *L. plantarum* – 3, *L. paracasei* – 3, *L. rhamnosus* – 1, *L. amylovorus −*2, *L. kitasatonis*/*L. amylovorus* – 1 *L. farciminis* – 2, *L. saerimneri*- 2 and *L. mucosae* – 2 strains (Table 2).

### Discrimination of reference *Lactobacillus* strains by ARDRA

ARDRA was first applied to 23 reference strains representing 19 species to verify whether the method sufficiently discriminates among species of the *Lactobacillus* genus. None of the 6 restrictions enzymes applied differentiated all 19 *Lactobacillus* species. The greatest discriminatory power was noted for the enzymes *Mse*I and *Hinf*I, as 14 different genotypes were obtained using these enzymes. *Alu*I and *Mbo*I each generated 12 unique patterns. *Hae*III, *Mps*I and *Taq*I digestion yielded 11, 9 and 7 specific patterns, respectively, among the reference lactobacilli (Table 3).

**Table 3:** Genotypes of reference *Lactobacillus* strains obtained via digestion of 16S rDNA amplicons using different restriction endonucleases; reference numbers of strains as shown in Table 1

No. of genotypes	*Mse*I	*Mbo*I	*Hinf*I	*Alu*I	*Hae*III	*Msp*I	*Taq*I
1	*L. salivarius* A1	*L. salivarius* A1	*L. salivarius* A1	*L. salivarius* A1	*L. salivarius* A1	*L. salivarius* A1	*L. salivarius* A1
	*L. salivarius* A2	*L. salivarius* A2	*L. salivarius* A2	*L. salivarius* A2	*L. salivarius* A2	*L. salivarius* A2	*L. salivarius* A2
	*L. agilis*		*L. agilis*		*L. agilis*		
2	*L. saerimneri*	*L. saerimneri*	*L. saerimneri*	*L. agilis*	*L. saerimneri*	*L. oris*	*L. farciminis*
3	*L. plantarum*	*L.agilis*	*L. ingluviei* R1	*L. saerimneri*	*L. plantarum*	*L. plantarum*	*L. ingluviei* R1
			*L. ingluviei* R2		*L. farciminis*	*L. farciminis*	*L. ingluviei* R2
			*L. reuteri* T1			*L. mucosae*	*L. oris*
							*L. acidophilus*
							*L. crispatus*
							*L. kitasatonis*
							*L. amylovorus*
							*L. gallinarum*
4	*L. farciminis*	*L. farciminis*	*L. farciminis*	*L. plantarum*	*L. johnsonii*	*L. reuteri* T1	*L. reuteri* T1 *L. reuteri* T2
5	*L. zeae*	*L. zeae*	*L. zeae*	*L. zeae*	*L. zeae*	*L. zeae*	*L. zeae*
	*L. rhamnosus*	*L. rhamnosus*	*L. casei*	*L. rhamnosus*	*L. rhamnosus*	*L. rhamnosus*	*L. rhamnosus*
	*L. casei*	*L. casei*	*L. paracasei*	*L. casei*	*L. casei*	*L. casei*	*L. casei*
	*L. paracasei*			*L. paracasei*	*L. paracasei*	*L. paracasei L. agilis*	*L. paracasei*
						*L. saerimneri*	*L. agilis*
							*L. saerimneri*
							*L. plantarum*
6	*L. johnsonii*	*L. gasseri*	*L. gasseri*	*L. gasseri*	*L. gasseri*	*L. gasseri*	*L. gasseri*
		*L. johnsonii*	*L. johnsonii*	*L. johnsonii*		*L. johnsonii*	*L. johnsonii*
7	*L. ingluviei* R1	*L. paracasei*	*L. mucosae*	*L. farciminis*	*L. mucosae*	*L. reuteri* T2	*L.mucosae*
	*L. ingluviei* R2						
	*L. mucosae*						
8	*L. acidophilus*	*L. acidophilus*	*L. acidophilus*	*L. acidophilus*	*L. acidophilus*	*L. acidophilus*	
		*L. crispatus*			*L. crispatus*	*L. crispatus*	
		*L. kitasatonis*			*L. kitasatonis*	*L. kitasatonis*	
		*L. amylovorus*			*L. amylovorus*	*L. amylovorus*	
		*L. gallinarum*			*L. gallinarum*	*L. gallinarum*	
9	*L. reuteri* T2	*L. reuteri* T1	*L. oris*	*L. ingluviei* R1	*L. ingluviei* R1	*L. ingluviei* R1	
				*L. ingluviei* R2	*L. ingluviei* R2	*L. ingluviei* R2	
10	*L. reuteri* T1	*L. reuteri* T2	*L. gallinarum*	*L. reuteri* T1	*L. reuteri* T1		
	*L. oris*			*L. reuteri* T2	*L. reuteri* T2		
				*L. mucosae*			
11	*L. gasseri*	*L. plantarum*	*L. rhamnosus*	*L. oris*	*L. oris*		
12	*L. amylovorus*	*L. ingluviei* R1	*L. crispatus*	*L. crispatus*			
	*L. gallinarum*	*L. ingluviei* R2	*L. kitasatonis*	*L. kitasatonis*			
		*L.mucosae*	*L. amylovorus*	*L. amylovorus*			
		*L. oris*		*L. gallinarum*			
13	*L. crispatus*		*L. reuteri* T2				
14	*L. kitasatonis*		*L. plantarum*				

Digestion of the 16S rDNA amplicon by most of the enzymes made it possible to distinguish such closely related species as *L. ingluviei*, *L. mucosae*, *L. reuteri* and *L. oris*, while only single enzymes differentiated *L. crispatus* from *L. kitasatonis* and species of the *L. casei* group. On the basis of analysis of genotypes obtained for the reference strains, the four enzymes with the greatest discriminatory power, *Mse*I, *Alu*I, *Hinf*I and *Mbo*I, were selected to differentiate the wild-type isolates. The combined use of three of these enzymes, *Mse*l, *Hinf*I and *Mbo*I, makes it possible to distinguish each species of *Lactobacillus* except for *L. casei* and *L. zeae*. However, because only *Mbo*I was capable of discriminating between *L. salivarius* and *L. agilis*, and only *Hinf*I differentiated *L. ingluviei* from *L. mucosae*, in order to confirm the differentiation and to increase the precision of the results obtained, the enzyme *Alu*I was additionally used.

**Fig. 1:**
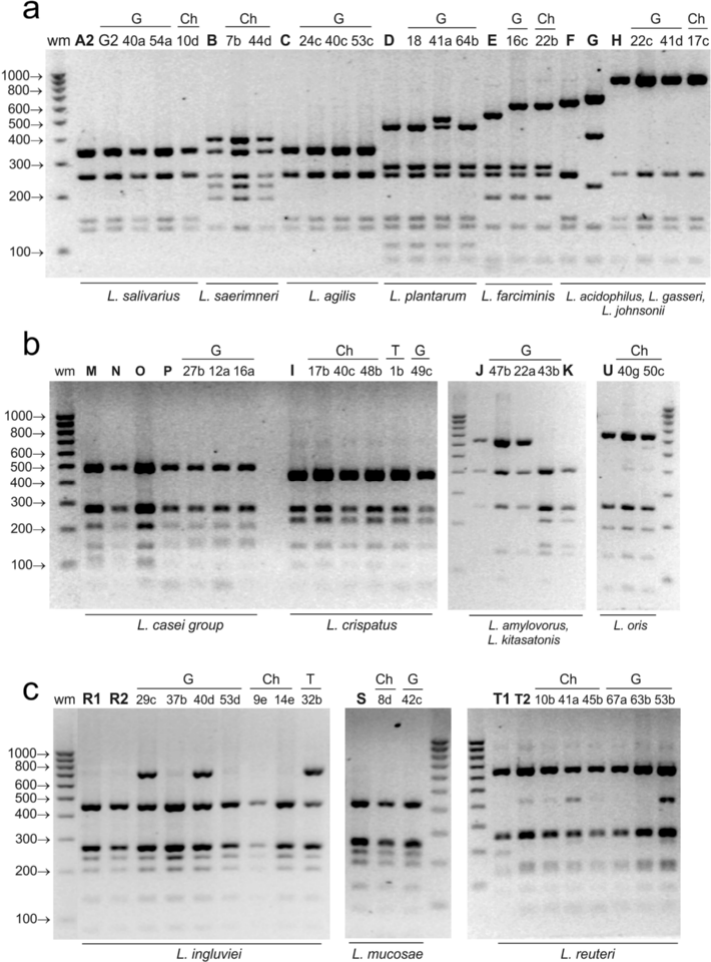
ARDRA patterns of reference and representative poultry *Lactobacillus* strains obtained by digestion of 16S rDNA amplicons with ***Mse*** **I**; restriction fragments were separated in 3 % agarose gel. Panel **a** - profiles of *L. salivarius* group strains, *L. plantarum*,* L. farciminis* and *L. delbruckii *group strains; panel **b** - profiles of *L. delbruckii* group strains and *L. oris* strains; panel **c** - profiles of *L. reuteri *group strains;  wm – DNA weight marker; bold letters A-T – reference strains as shown in Table 1; A2 - *L. salivarius*, B - *L. saerimneri*, C - *L. agilis*, D – *L. plantarum*, E – *L. farciminis*, F – *L. acidophilus*, G – *L. gasseri*, H – *L. johnsonii*, I – *L. crispatus*, J – *L. amylovorus*, K – *L. kitasatonis*, M – *L. casei*, N – *L. rhamnosus*, O – *L. zeae*, P - *L. paracasei*, R1 and R2 *– L. ingluviei,* S *– L. mucosae,* T1 and T2 – *L. reuteri,* U – *L. oris*; G, Ch, T – strains isolated from geese, chickens and turkeys, respectively

*Mse*I was the only enzyme to differentiate the species *L. crispatus*, *L. kitasatonis* and *L. amylovoru/L. gallinarum.* Another advantage of this enzyme is its ability to discriminate between closely related species, i.e., to distinguish *L. gasseri* from *L. johnsonii* and *L. crispatus* from *L. acidophilus*. However, unlike most of the enzymes tested (*Alu*I, *Hae*III, *Mps*I, *Tag*I and *hindi*), the use of *Mse*I did not enable differentiation of *L. ingluviei* from *L. mucosae* or *L. reuteri* from *L. oris* (Fig. 1, Table 3).

**Fig. 2:**
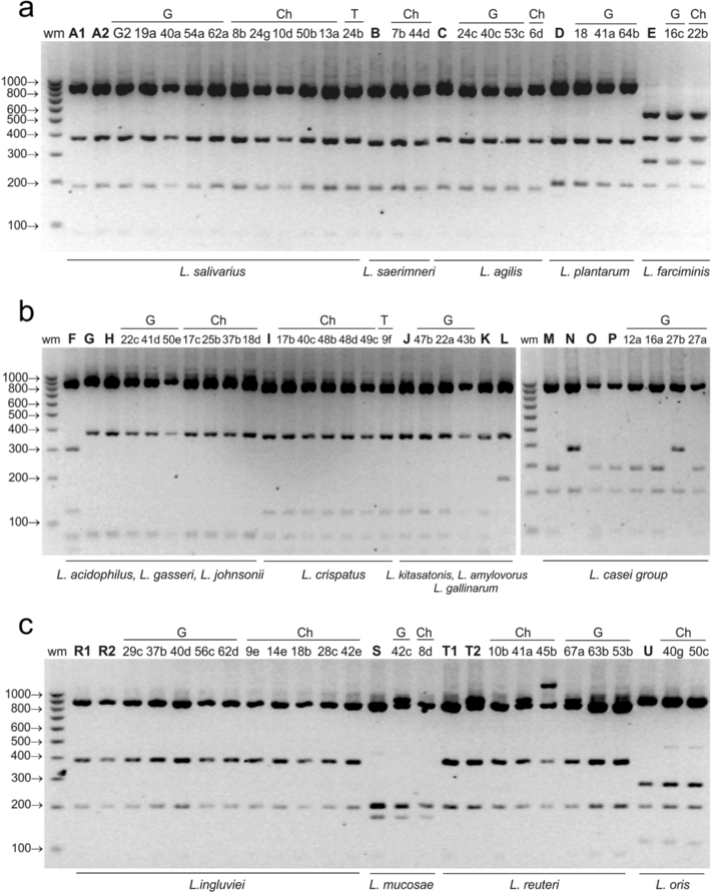
ARDRA patterns of reference and representative poultry *Lactobacillus* strains obtained by digestion of 16S rDNA amplicons with ***Hinf*** **I**; restriction fragments were separated in a 3 % agarose gel. Panel **a** - profiles of the *L. salivarius* group strains, *L. plantarum* and *L. farciminis*; panel **b** - profiles of the strains belonging to the *L. delbruckii* and *L. casei* groups; panel **c** - profiles of *L. reuteri *group strains; wm – DNA weight marker; bold letters A-T – reference strains as shown in Table 1; A1 and A2 - *L. salivarius*, B - *L. saerimneri*, C - *L. agilis*, D – *L. plantarum*, E – *L. farciminis*, F – *L. acidophilus*, G – *L. gasseri*, H – *L. johnsonii*, I – *L. crispatus*, J – *L. amylovorus*, K – *L. kitasatonis*, L – *L. gallinarum*, M – *L. casei*, N – *L. rhamnosus*, O – *L. zeae*, P - *L. paracasei*, R1 and R2 *– L. ingluviei,* S *– L. mucosae,* T1 and T2 – *L. reuteri,* U – *L. oris*; G, Ch, T – strains isolated from geese, chickens and turkeys, respectively

*Hinf*I was the only enzyme to differentiate *L. rhamnosus* from the remaining species of the *L. casei* group, as well as *L. amylovorus* from *L. gallinarum*. Moreover, like *Mse*I, *Hinf*I enabled differentiation of the closely related species *L. crispatus* and *L. acidophilus* (*L. delbruckii* group) (Fig. 2).

**Fig. 3:**
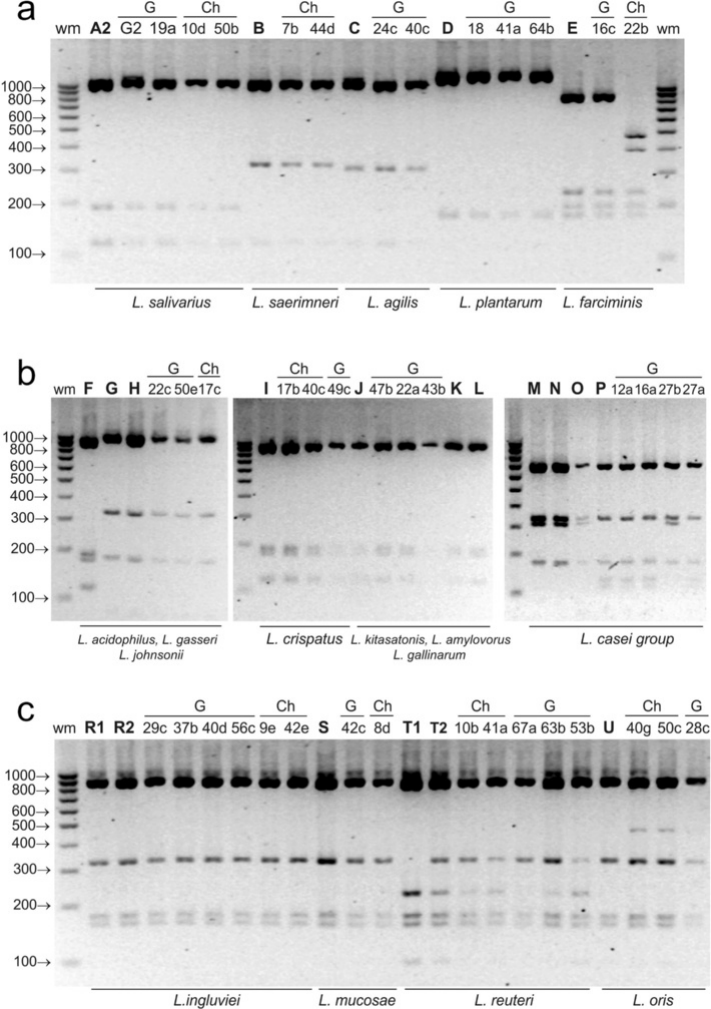
ARDRA patterns of reference and representative poultry *Lactobacillus* strains obtained by digestion of 16S rDNA amplicons with ***Mbo*** **I**; restriction fragments were separated in a 3 % agarose gel. Panel **a** - profiles of *L. salivarius* group strains, *L. plantarum* and *L. farciminis*; panel **b** - profiles of the strains belonging to the group of *L. delbruckii* and *L. casei*; panel **c** - profiles of *L. reuteri *group strains; wm – DNA weight marker; bold letters A-T – reference strains as shown in Table 1; A2 - *L. salivarius*, B - *L. saerimneri*, C - *L. agilis*, D – *L. plantarum*, E – *L. farciminis*, F – *L. acidophilus*, G – *L. gasseri*, H – *L. johnsonii*, I – *L. crispatus*, J – *L. amylovorus*, K – *L. kitasatonis*, L – *L. gallinarum*, M – *L. casei*, N – *L. rhamnosus*, O – *L. zeae*, P - *L. paracasei*, R1 and R2 *– L. ingluviei,* S *– L. mucosae,* T1 and T2 – *L. reuteri,* U – *L. oris*; G, Ch, T – strains isolated from geese, chickens and turkeys, respectively

The use of the enzyme *Mbo*I enabled differentiation of *L. paracasei* from the remaining species of the *L. casei* group. In the profile of *L. paracasei* two additional bands of 118 and 130 bp were present, while one band of 312 bp occurring in the profiles of *L. casei*, *L. zeae* and *L. rhamnosus* was absent (Fig. 3b).

**Fig. 4:**
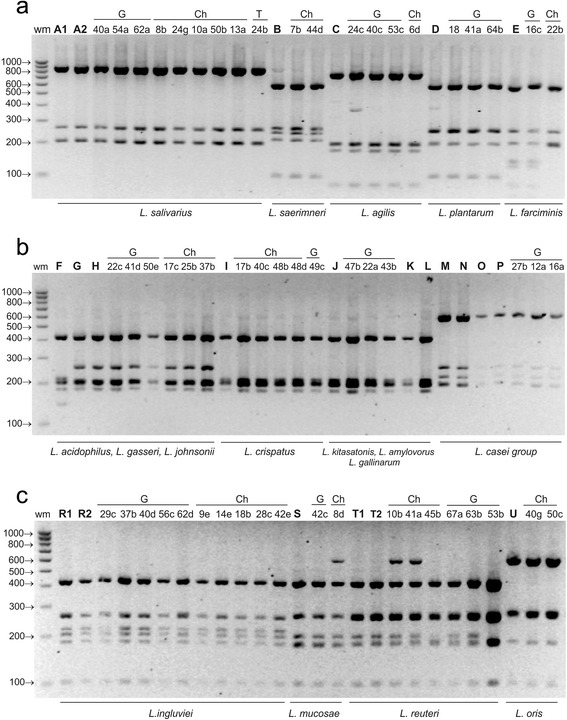
ARDRA patterns of reference and representative poultry *Lactobacillus* strains obtained by digestion of 16S rDNA amplicons with ***Alu*** **I**; restriction fragments were separated in a 3 % agarose gel. Panel **a** - profiles of *L. salivarius* group strains, *L. plantarum* and *L. farciminis*; panel **b** - profiles of the strains belonging to the group of *L. delbruckii* and *L. casei*; panel **c** - profiles of *L. reuteri* group strains ; wm – DNA weight marker; bold letters A-T – reference strains as shown in Table 1; A1 and A2 - *L. salivarius*, B - *L. saerimneri*, C - *L. agilis*, D – *L. plantarum*, E – *L. farciminis*, F – *L. acidophilus*, G – *L. gasseri*, H – *L. johnsonii*, I – *L. crispatus*, J – *L. amylovorus*, K – *L. kitasatonis*, L – *L. gallinarum*, M – *L. casei*, N – *L. rhamnosus*, O – *L. zeae*, P - *L. paracasei*, R1 and R2 *– L. ingluviei,* S *– L. mucosae,* T1 and T2 – *L. reuteri,* U – *L. oris*; G, Ch, T – strains isolated from geese, chickens and turkeys, respectively

The main advantage of *Alu*I was the ability to distinguish *L. salivarius* from *L. agilis* and *L. ingluviei* from *L. mucosae* (Fig. 4).

### Discrimination of wild *Lactobacillus* isolates by ARDRA

By analysing the electrophoresis profiles of restriction fragments obtained using *Mse*I, *Alu*I, *Hinf*I and *Mbo*I, 80 wild poultry isolates with rod-shaped morphology were classified into 15 *Lactobacillus* species belonging to 6 phylogenetic groups. Sixteen isolates have restriction profiles characteristic for *L. salivarius*, 12 for *L. johnsonii*, 11 for *L. ingluviei*, 8 for *L. crispatus*, 8 for *L. reuteri*, 4 for *L. agilis*, 4 for *L. oris*, 3 for *L. plantarum*, 3 for *L. paracasei*, 2 for *L. farciminis*, 2 for *L. saerimneri*, 2 for *L. amylovorus*, 2 for *L. mucosae*, 1 for *L. kitasatonis* and 1 for *L. rhamnosus* (Table 2, Figs. 1, 2, 3 and 4). Strains of the species *L. plantarum*, *L. paracasei*, *rhamnosus*, *L. amylovorus and L. kitasatonis* were isolated only from geese, and *L. saerimneri* only from chickens. Other *Lactobacillus* species were present in different species of birds.

The electrophoretic profiles of 16S rDNA digested with endonucleases contained several (2–8) restriction fragments ranging from 80 to 1300 bp. The sizes of all bands characteristic for different *Lactobacillus* species are shown in Table 4.

**Table 4:** Sizes (bp) of restriction fragments obtained by cleavage of 16S rDNA amplicons of *Lactobacillus* strains isolated from poultry. The values in brackets refer only some strains

Group	No. of isolates	Identification	Size (bp) of restriction fragments found in wild strains			
			*Mse*I	*Hinf*I	*Mbo*I	*Alu*I
*L. salivarius*	17	*L. salivarius*	350, 256, 145, 130	900, 385, 190	1070, 190, 118	840, 265, 205
	2	*L. saerimneri*	400, 350, 256, 237, 202, 130	900, 385, 190	1070, 324	570, 270, 250, 210, 97
	4	*L.agilis*	350, 256, 145, 130	900, 385, 190	1070, 312	720, (363), 205, 180, 80
*L. plantarumm*	3	*L. plantarum*	(520), 470, 295, 256, 145, 130, 110, 90	910, 385, 190	1300, 175	570, 265, 205, 97
*L. alimentarius*	2	*L. farciminis*	620, 295, 256, 202, 130, 90	540, 390, 300, 200	(850), (470), (395), 235, 200, 175,	570, 265, 205, (130), (111), (80)
*L. reuteri*	11	*L. ingluviei*	(650), 450, 256, 237, 202, 130, 90	900, 385, 200	850, 324, 180, 165	410, 265, 210, 205, 180, 100
	2	*L. mucosae*	450, 256, 237, 202, 130, 90	(910), 900, 200, 170	850, 324, 180, 165	(570), 410, 265, 205, 180, 100
	8	*L. reuteri*	650, (450), 256, 145, 130, 90	(1170), (910), 900, 385, 200	850, 324, 180, 165	(570), 410, 265, 205, 180, 100
	4	*L. oris*	650, (550), (430), 256, 202, 130, 90	910, 290, 200, 110	850, (480), 324, 180, 165	570, 270, 180, 100
*L. delbruckii*	12	*L. johnsonii*	940, 256, 145, 130, 90	910, 385, 85	920, 324, 180	410, 250, 205, 180
	8	*L. crispatus*	450, 256, 237, 130, 90	900, 385, 120, 85	920, 190, 175, 118	410, 210, 205, 180
	2	*L. amylovorus*	650, 450, 256, 130, 90	900, 385, 120, 85	920, 190, 175, 118	410, 210, 205, 180
	1	*L. kitasatonis*	450, 256, 237, 145, 130, 90	900, 385, 120, 85	920, 190, 175, 118	410, 210, 205, 180
*L. casei*	3	*L. paracasei*	470, 256, 202, 130, 110, 90	910, 290, 200	700, 330, 175, 130, 118	570, 265, 230, 205
	1	*L. rhamnosus*	470, 256, 202, 130, 110, 90	910, 385, 200	700, 330, 312, 175	570, 265, 230, 205
Total: 80						

The electrophoretic patterns of the wild-type isolates classified as *L. salivarius*, *L. saerimneri*, *L. crispatus*, *L. kitasatonis*, *L. amylovorus*, *L. johnsonii*, *L. paracasei* and *L. rhamnosus* were identical to those of the reference strains in the case of each of the restriction enzymes applied. The profiles of the isolates characteristic for the remaining *Lactobacillus* species, i.e., *L. agilis*, *L. reuteri*, *L. ingluviei*, *L. mucosae*, *L. oris*, *L. plantarum* and *L. farciminis*, were not always identical to the electrophoretic patterns of the reference strains. The differences usually involved one band and were observed even in the profiles of reference strains belonging to the same species, i.e., *L. reuteri*.

Differences between the restriction profiles of strains characteristic for *L. reuteri* appeared in the case of each of the restriction enzymes used (Figs. 1c, 2c, 3c and 4c). In *Mse*I-ARDRA, in some of the wild-type strains and in the profile of one of the two reference strains of *L. reuteri* (LMG 18238) there was a band of 450 bp, while in the profile of *L. reuteri* strain LMG 9213 an additional band of low intensity, 202 bp in size, was observed (Fig. 1c). In the profiles obtained following digestion with *Hinf*I, there was an additional product of 910 bp in some isolates and in the reference strain *L. reuteri* LMG 18238, and an additional band of 1200 bp in the profile of strain 45b (Fig. 2c). In *Mbo*I-ARDRA analysis the occurrence of a band of 324 bp was observed in all strains of *L. reuteri* except for the reference strain *L. reuteri* LMG 18238. In the case of isolates classified as *L. ingluviei* differences were observed between the profiles obtained using *Mse*I restriction – in some isolates an additional fragment of 650 bp was present. Differences involving single bands of low intensity were observed among strains classified as *L. oris* (Figs. 1b, 2c and 3c), *L. mucosae* (Figs. 2c and 4c) and *L. plantarum* (Fig. 1a).

### Genetic diversity of the examined strains

**Fig. 5:**
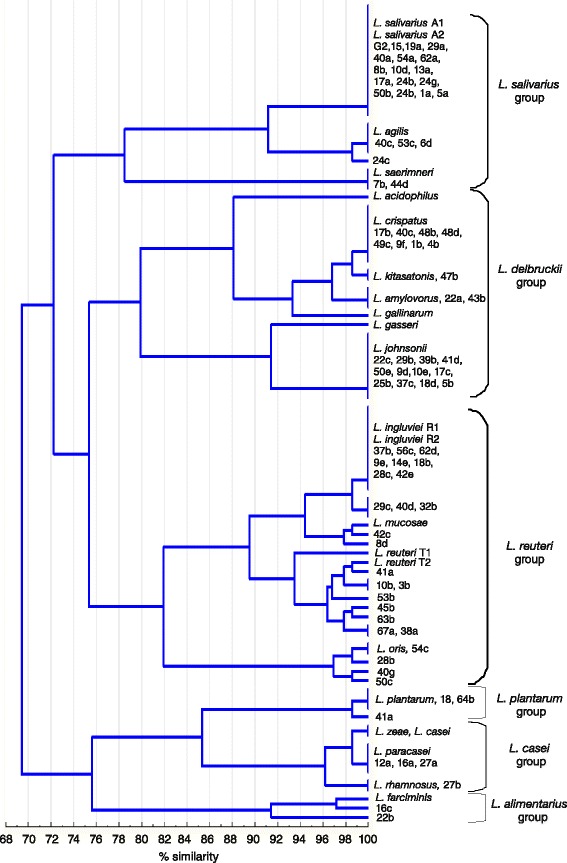
Dendrogram based on UPGMA clustering of combined *Mse*I, *Hinf*I, *Mbo*I and *Alu*I electrophoretic patterns obtained from *Lactobacillus* reference and wild strains; reference strains as shown in Table 1

Restriction fragments obtained with *Mse*I, *Hinf*I, *Mbo*I and *Alu*I were used to determine the genetic diversity of the examined lactobacilli and to cluster them into specific groups. These results can be seen in the dendrogram generated using the UPGMA clustering algorithm and percent disagreement as a genetic distance (Fig. 5). All strains are clustered at a similarity level of 69 %, which could be considered evidence of a homogenous population of one genus. All wild strains exhibited high similarity, ie. ≥91.5 % to the appopriate reference strains. Restriction profiles of 60 (75 %) isolates were identical (100 % similarity) to the profiles of reference strains.

At a similarity level of 69 % the strains formed two main clusters. One cluster consisted of strains with profiles characteristic for the *L. casei* group, *L. plantarum* and *L. farciminis*, while all the other strains analysed were in the second complex cluster. At a similarity level 76 % five clusters were formed: 1) the strains of the *L. salivarius* group (*L. salivarius*, *L. agilis* and *L. saerimneri*) 2) the strains of the *L. delbdruckii* group (*L. acidophilus*, *L. johnsonii*, *L. gasseri*, *L. crispatus*, *L. kitasatonis*, *L. amylovoru* and *L. gallinarum*), 3) the strains of the *L. reuteri* group (*L. ingluviei*, *L. mucosae*, *L. reuteri* and *L. oris*), 4) the strains of the *L. casei* group (*L. casei*, *L. paracasei*, *L. zeae* and *L. rhamnosus*) and the *L. plantarum* group and 5) the strains of the *L. alimentarius* group (*L. farciminis*).

At a similarity level of 100 %, 36 genotypes were distinguished among the 23 reference strains and 80 field isolates (Fig. 5). The greatest distance was noted among strains characteristic for *L. farciminis*, which were grouped at a similarity level of 91.5 %, and among strains classified as *L. reuteri*, forming a common clade at a similarity value of 93.5 %.

ARDRA using the four restriction enzymes enabled differentiation of all the *Lactobacillus* species analysed except for *L. zeae* and *L. casei*.

### Identification by sequencing of the 16S rRNA gene

rDNA sequencing of all isolates selected for analysis revealed 99–100 % homology to the sequences of the reference *Lactobacillus* strains deposited in GenBank (Table 5). The test confirmed the species identification of the isolates that was previously established using the ARDRA technique. The results obtained are particularly significant in the case of strains whose restriction profiles were not always (not in the case of all restriction enzymes) identical to those of the reference strains, i.e., 42c-*L. mucosae* (Fig. 2c), 29c-*L. ingluviei* (Fig. 1c), 22b-*L. farciminis* (Figs. 1a and 3a), 41a-*L. reuteri* (Fig. 4c) and 45b-*L.reuteri* (Fig. 2c).

**Table 5:** Identification of representative *Lactobacillus* strains by 16S rRNA gene sequence analysis compared to results obtained by 16S-ARDRA and MALDI-TOF MS

Isolate	Source	Identification by 16S rDNA sequence	Identity value	GenBank accession no.	Identification by 16S-ARDRA (% similarity between wild and reference strains, based on Fig. 5)	Identification by MALDI-TOF MS, log(score)
10d	chicken	*L. salivarius*	99 %	KR492877	*L. salivarius* (100 %)	*L. salivarius* 2.162
17c	chicken	*L. johnsonii*	99 %	KR492880	*L. johnsonii* (100 %)	*L. johnsonii* 1.971
						*L. gasseri* 1.770
9e	chicken	*L. ingluviei*	99 %	KR492878	*L. ingluviei* (100 %)	*L. ingluviei* 2.182
29c	goose	*L. ingluviei*	99 %	KR492882	*L. ingluviei* (98.5 %)	*L. ingluviei* 2.018
22b	chicken	*L. farciminis*	99 %	KR492881	*L. farciminis* (91.5 %)	*L. farciminis* 1.88
41a	chicken	*L. reuteri*	99 %	KR492883	*L. reuteri* (93.5 %)	*L. reuteri* 2.026
45b	chicken	*L. reuteri*	99 %	KR492885	*L. reuteri* (93.5 %)	*L. reuteri* 1.900
42c	goose	*L. mucosae*	100 %	KR492884	*L. mucosae* (99 %)	*L. mucosae* 2.121

### Comparison of MALDI-TOF MS and ARDRA identification results

In the case of MALDI-TOF technique the univocal results in the range log(score) 1.7-3.0, were obtained for 67 (83.75 %) strains. For the remaining 13 (16.25 %) strains identification was considered unreliable due to the lack of reproducibility in the two best matches that showed similar (difference >0.2) log(score) values. Using ARDRA technique made it possible to identify to species level of all 80 strains tested. Taking into account an ambiguous results (16.25 %) MALDI-TOF MS methodology yielded clear agreement identification of 67 (83.75 %) of 80 isolates identified by ARDRA.

The use of ARDRA method and 16S rDNA sequencing eliminated doubts as to the correct classification of 16.25 % strains for which non conclusive identification result had been obtained in MS. ARDRA revealed that the 12 strains which were identified in MS as *L. johnsonii*/*L. gasseri* belonged to the species of *L. johnsonii*, and one strain marked as *L. kitasatonis*/*L. amylovorus* was identified as *L. kitasatonis.* Notable is the fact that for those 16.25 % samples the first best match from Biotyper databse was always consistent with the identification achieved by 16S-ARDRA.

Both methods do not allow to distinguish *L. casei* from *L. zeae*, but both enable the discrimination other species of *L. casei* group, ie. *L. paracasei* and *L. rhamnosus*.

16S rDNA analysis also showed that the MS species identification results with intermediate values for the log(score) (1.70–1.99) were correct (for 24 isolates, including 6 for which the two best matches with similar log(score) values indicating different species).

In comparison with MALDI-TOF MS, ARDRA is more labour-intensive, but more reliable methods that allow for differentiation even close related *Lactobacillus* species. In the cases of some species restriction analysis it revealed intraspecific differences.

## Discussion

In this study we evaluated the usefulness of MALDI-TOF MS and 16S-ARDRA for identification of *Lactobacillus* isolates from poultry. The techniques proved to be valuable tools for species-level classification of lactobacilli and yielded comparable results.

The *Lactobacillus* bacteria we identified belonged to 15 species. Strains of the species *L. salivarius* (21.25 %), *L. johnsonii* (15 %) and *L.ingluviei* (13.75 %) were predominated.

For 83.75 % of the isolates both methods produced concordant unequivocal results, and in the case of the remaining 16.25 % of isolates, whose identification was considered unreliable due to the lack of reproducibility in the two best matches showing similar log(score) values, the best match generated by Biotyper was always consistent with the identification by 16S-ARDRA. Other studies report similar high-percentage agreement between mass spectrometry and different genotypic methods, including 16S rDNA sequencing [22], 16S-ARDRA [24], analysis of the region 16S-23S of rDNA [3] and species-specific PCR [28].

In recent years, MALDI-TOF MS has emerged as a promising and reliable tool for bacteria identification [29], including lactobacilli isolated from diary and meat products [24, 30, 31], carious dentin in children [28], human oral cavities and women’s vaginas [22] and poultry [3]. MALDI-TOF MS is quick and cost-effective and allows many samples to be pooled in one analysis. The performance of the method depends on many factors, among which sample preparation plays a key role. It is especially important in the case of Gram-positive bacteria, for which intact-cell MALDI-TOF MS somethimes generates poor spectra due to their thick peptidoglycan cell walls. With this in mind, in this study we used an on-plate extraction method that is intermediate between two well-described methods, i.e., the direct colony method and the standard protein extraction method. Formic acid overlaid directly onto the bacterial smear in the on-plate extraction method facilitates cell wall disruption, yielding better spectra and identification results with higher log(score) values as compared to the intact cell method [26, 27]. At the same time, the analysis time is much shorter than in the case of standard extraction, which involves preparing a suspension of bacteria in alcohol and 3 centrifugation steps.

Using the on-plate extraction method for 30 % of the wild *Lactobacillus* strains we obtained results with intermediate log(scores) of 1.70–1.99, which indicate correct genus identification but only presumptive species identification. In addition, the reliability of the identification of some of these strains was further reduced by the lack of conformity between the first and the second best match and/or because the log(score) of the second match (compatible or incompatible with the first match) was less than 1.70. On the other hand, for all strains with identification log(scores) 1.70–1.99 the best match indicated the same species as 16S-ARDRA, suggesting that despite the low log(score) the MALDI-TOF MS identification to the species level was correct. Other authors [3, 22] have also observed frequent occurrence of Biotyper log(scores) ≤1.99 in identifying *Lactobacillus* bacteria using MALDI-TOF MS (intact-cell or standard extraction method) as well as agreement between the results of such values and the results of species identification obtained in genetic methods (16S rDNA sequencing or analysis of the region 16S-23S).

The second weak point of the MALDI-TOF MS technique demonstrated in the present study is its inability to reliably differentiate (despite log(score) values ≥2.00) closely related species, such as *L. johnsonii* and *L. gasseri*, *L. amylovorus* and *L. kitasatonis*, or lactobacilli of the *L. casei* group. The same problem has been reported in our previous work [3] as well as in studies by Dušková et al. [24], Sedo et al. [32] and Schulthess et al. [26]. It seems that expanding the commercial database by generating one’s own reference spectra may improve rates of species identification [26].

The ARDRA technique used in the present study for the identification of *Lactobacillus* isolated from geese, chickens and turkeys, proved to be highly discriminatory. The combined application of several restriction enzymes, i.e., *Mse*I, *Hinf*I, *Mbo*I and *Alu*I, for digestion of 16S rDNA allowed us to divide the 80 isolates into several phylogenetic groups and to affiliate them to 15 species. Strains of the species *L. salivarius* predominated in all species of birds examined. Bacteria of the species *L. johnsonii, L. ingluviei, L. crispatus* and *L. reuteri* were also frequently isolated. The results of the present study are in agreement with previous observations that *L. salivarius*, *L. johnsonii* and *L. ingluviei* are the predominant *Lactobacillus* sp. In the GIT of geese [3], and *L. crispatus*, *L. reuteri and L. salivarius* are the most abundant intestinal lactobacilli in chickens [2, 33, 34].

The 16S-ARDRA technique we used to identify lactobacilli is an alternative to more laborious and expensive methods for the identification of eubacteria, as it is simple, relatively fast and highly repetitive. It analyses only one gene, i.e., the gene encoding 16S rRNA, using universal primers and the same PCR and digestion conditions for all *Lactobacillus* species. The discriminatory power of ARDRA depends on the correct choice of restriction endonucleases.

Species identification of poultry lactobacilli determined on the basis of 16S-ARDRA was confirmed by MALDI-TOF MS and 16S rDNA sequencing of representative strains.

The effectiveness of ARDRA observed in our study for species identification of bacteria of the genus *Lactobacillus* has also been reported by other authors. 16S-ARDRA has previously been used successfully for identification of lactobacilli isolated from dairy and meat products [21], wine [35], mothers’ stool and breast milk and infants’ stool [36], intestines of calves [37] and women’s vaginas [38]. Some authors, however, have demonstrated that ARDRA is incapable of discriminating species with high 16S rDNA sequence homology. Rodas et al. [35], who identified LAB by 16S-ARDRA using *Bfa*I and *Mse*I, were unable to distinguish the species of the *L. casei* group, *L. reuteri* from *L. oris* or *L. plantarum* from *L. pentosus*. Difficulty in differentiating species of the *L. casei* group using 16S-ARDRA has been also reported by Ksicova et al. [21]. The restriction enzymes used by these authors, *Alu*I and *Msp*I, also failed to distinguish between *L. plantarum* and *L. paraplantarum* and between *L. johnsonii* and *L. gasseri*. Our study indicates that the problems in distinguishing between closely related species may be solved in most cases by the use of appropriately selected restriction enzymes. We confirmed that *L. johnsonii* cannot be distinguished from *L. gasserii* using *Alu*I or *Msp*I, but the use of other restriction enzymes, i.e., *Mse*l and *Hae*III, enabled discrimination of these species. We also obtained a positive effect of differentiation by the ARDRA protocol in the case of other closely related species, i.e., *L. reuteri* and *L. oris* and species of the *L. casei* group, comprising *L. casei*, *L. paracasei*, *L. rhamnosus* and *L. zeae*. The use of the enzymes *Hinf*I and *Mbo*I enabled differentiation of *L. rhamnosus*, *L. paracasei* and *L.casei*/*L.zeae*. Unfortunately, none of the 7 restriction enzymes tested was able to distinguish *L. casei* from *L. zeae*.

The grouping of *Lactobacillus* strains (dendrogram) analysed by ARDRA is congruent with the evolutionary distance of the 16S rRNA gene sequences and reflects the actual genetic relationship between the strains. Based on their 16S rDNA sequence the *Lactobacillus* species are currently divided into 15 large phylogenetic groups, 4 pairs (small phylogenetic groups containing only two species) and 10 groups represented by single species [38, 39]. The tested isolates of poultry origin belonged to 6 large phylogenetic groups, i.e., *L. salivarius* (identified species: *L. salivarius*, *L. saerimnerii* and *L. agilis*), *L. delbruckii* (*L. johnsonii*, *L. crispatus*, *L. amylovorus* and *L. kitasatonis*), *L. plantarum*, *L. alimenatrius* (*L. farciminis*), *L. reuteri* (*L. reuteri, L. ingluviei, L. mucosae* and *L. oris*) and *L. casei* (*L. paracasei* and *L. rhamnosus*).

Our research has shown that ARDRA not only differentiates strains well at the species level, but may also reflect differences within a species. The differences we observed in the electrophoretic profiles of the restriction fragments of strains identified as the same *Lactobacillus* species usually involved one band. Such differences occurred between profiles of the reference strains *L. reuteri* LMG 9213 and *L. reuteri* LMG 18238, as well as among field isolates of some *Lactobacillus* species. Intraspecific differentiation of *Lactobacillus* strains analysed using 16S-ARDRA has also been observed by other authors [21, 35].

## Conclusions

In conclusion, our study demonstrates that the MALDI-TOF MS and 16S-ARDRA assays are valuable tools for the identification of avian lactobacilli to the species level. The major advantages of MALDI-TOF MS are its rapidity, simplicity and low cost. Despite generating a high percentage (30 %) of log(score) results <2.00, the on-plate extraction method is characterized by high-performance and agreement of strain identification with that obtained by 16S-ARDRA. For strains for which the two best Biotyper matches of log(sore) ≥ 1.7 indicate various species (eg. *L. johnsonii* and *L. gasseri*) and the difference between their log(score) values is <0.30, MALDI-TOF must be used in combination with genotyping techniques to achieve an unequivocal outcome.

The developed ARDRA protocol can be used for discrimination of *Lactobacillus* bacteria from different habitats. The advantages of ARDRA over other methods for bacterial identification based on DNA analysis are its simplicity, reproducibility and lower cost. In addition, this technique allows us not only to identify the species, but also to determine the genetic relationships between strains. Due to its high power of discrimination, polymorphic ARDRA patterns may sometimes reflect differences between strains within a species. One limitation of ARDRA is its inability to discriminate between species of very high 16S rDNA sequence homology, i.e., *L. casei* and *L. zeae*.

## Abbreviations

16S-ARDRAA, Amplified Ribosomal DNA Restriction Analysis of 16S rDNA; MALDI-TOF MS, Matrix-assisted laser desorption/ionization time-of-flight mass spectrometry; MRS, de Man, Rogosa and Sharp; Nd-YAG, neodymium-doped yttrium aluminium garnet
